# Data on nucleoid-associated proteins isolated from *Mycoplasma Gallisepticum* in different growth phases

**DOI:** 10.1016/j.dib.2020.105853

**Published:** 2020-06-12

**Authors:** I. Zubov Aleksandr, A. Semashko Tatiana, V. Evsyutina Daria, G. Ladygina Valentina, I. Kovalchuk Sergey, H. Ziganshin Rustam, A. Galyamina Maria, Yu. Fisunov Gleb, V. Pobeguts Olga

**Affiliations:** a Federal Research and Clinical Center of Physical-Chemical Medicine of Federal Medical Biological Agency, Moscow, Russia; b Shemyakin-Ovchinnikov Institute of Bioorganic Chemistry, Moscow, Russia

**Keywords:** Mycoplasma gallisepticum, Synchronous growth, Nucleoid, Nucleoid-associated proteins, Proteomic analysis, Data-independent acquisition

## Abstract

*Mycoplasma gallisepticum* (MG) is one of the smallest free-living and self-replicating organisms, it is characterized by lack of cell wall and reduced genome size. As a result of genome reduction, MG has a limited variety of DNA-binding proteins and transcription factors. To investigate the dynamic changes of the proteomic profile of MG nucleoid, that may assist in revealing its mechanisms of functioning, regulation of chromosome organization and stress adaptation, a quantitative proteomic study was performed on MG nucleoids obtained from the cell culture in logarithmic and stationary phases of synchronous growth. MG cells were grown on a liquid medium with a 9 h starvation period. Nucleoids were obtained from the cell culture at the 26^th^ and the 50^th^ hour (logarithmic and stationary growth phases respectively) by sucrose density gradient centrifugation. LC-MS analysis was carried out on an Ultimate 3000 RSLCnano HPLC system connected to a Fusion Lumos mass spectrometer, controlled by XCalibur software (Thermo Fisher Scientific) via a nanoelectrospray source (Thermo Fisher Scientific). For comprehensive peptide library generation one sample from each biological replicate was run in DDA mode. Then, all the samples were run in a single LC-MS DIA run. Identification of DDA files and DIA quantitation was performed with MaxQuant and Skyline software, correspondingly. All raw data generated from IDA and DDA acquisitions are presented in the PRIDE database with identifier PXD019077.

Specifications table

**Table:** 

Subject	Biology
Specific subject area	Proteomics
Type of data	DIA LC-MS data, identification and quantification data
How data were acquired	Data-independent acquisition, data-dependent acquisition (DIA/DDA) using Fusion Lumos mass spectrometer.
Data format	Raw and analyzed data
Parameters for data collection	M. gallisepticum nucleoids were isolated by centrifugation in sucrose density gradient from bacteria in log and stationary states of synchronous culture growth.
Description of data collection	Data were obtained by mass spectrometric DIA measurements of nucleoid-containing fractions with iRT peptides for generation of a spectral library. For each sample type 3 biological replicates with from 2 to 3 technical sample preparation replicates were analyzed, in total 9 DDA and 32 DIA runs.
Data source location	Research and Clinical Center of Physical-Chemical Medicine, Moscow, Russian Federation
Data accessibility	Repository name: PRIDE Data identification number: PXD019077 https://www.ebi.ac.uk/pride/archive/projects/PXD019077

## Value of the data

• This dataset contains the first published detailed proteomic profiles of M. gallisepticum nucleoids obtained from the cell culture in different phases of synchronous growth.
• This data might be used to expand the knowledge on nucleoid-associated proteins in genome-reduced organisms.
• The data presented can be of value for the research on chromosome organization, mechanisms of functioning, regulation of and stress adaptation in Mycoplasmas.

## 1 Data description

*Mycoplasma gallisepticum* (MG) belongs to the class Mollicutes and is commonly involved in the chronic respiratory disease of avian species [1,2]. Being one of the smallest free-living and self-replicating organisms, it is characterized by lack of cell wall and reduced genome size [3]. As a result of genome reduction, MG has a limited variety of DNA-binding proteins (DBP) and transcription factors[4]. Along with the fact, that bacterial chromosome has to be compact enough to fit inside the small cell, it also has to preserve its accessibility to the bacterial replication, segregation, and transcription machinery. Among the many DBPs, nucleoid-associated proteins (NAP), small proteins that bind DNA with low specificity and can influence chromosome organization under changing environmental conditions, are involved in maintaining this highly organized and yet dynamic chromosome structure [5,6]. To investigate the dynamic changes of the proteomic profile of MG nucleoid, that may assist in revealing its mechanisms of functioning, regulation of chromosome organization and stress adaptation, a quantitative proteomic study was conducted in MG nucleoids obtained from the cells at 26th and 50th hours (logarithmic (LPSG) and stationary phases of synchronous growth (SPSG) respectively).

**Fig. 1:**
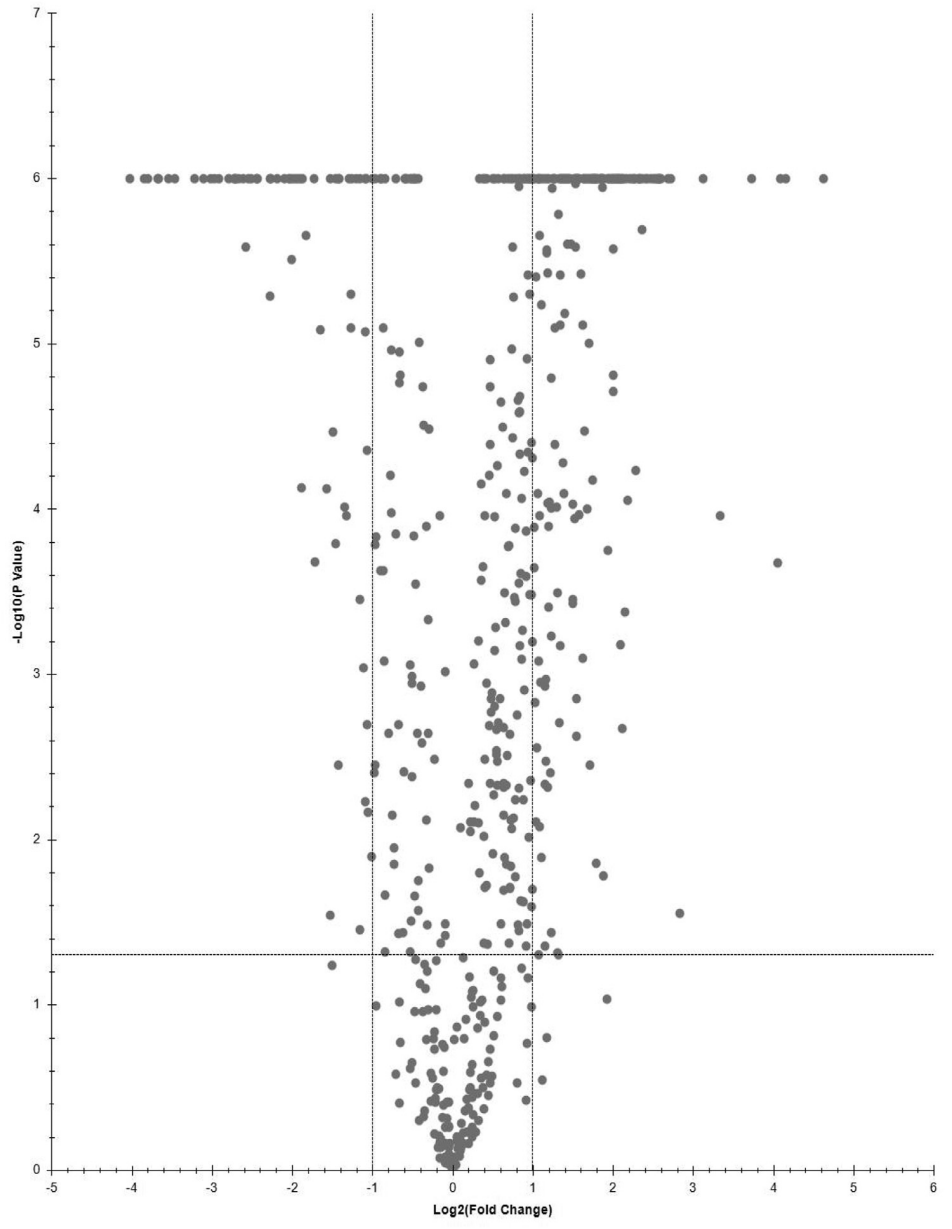
Quantitative proteomics data for cell culture in log state: nucleoid to cell lysate.

**Fig. 2:**
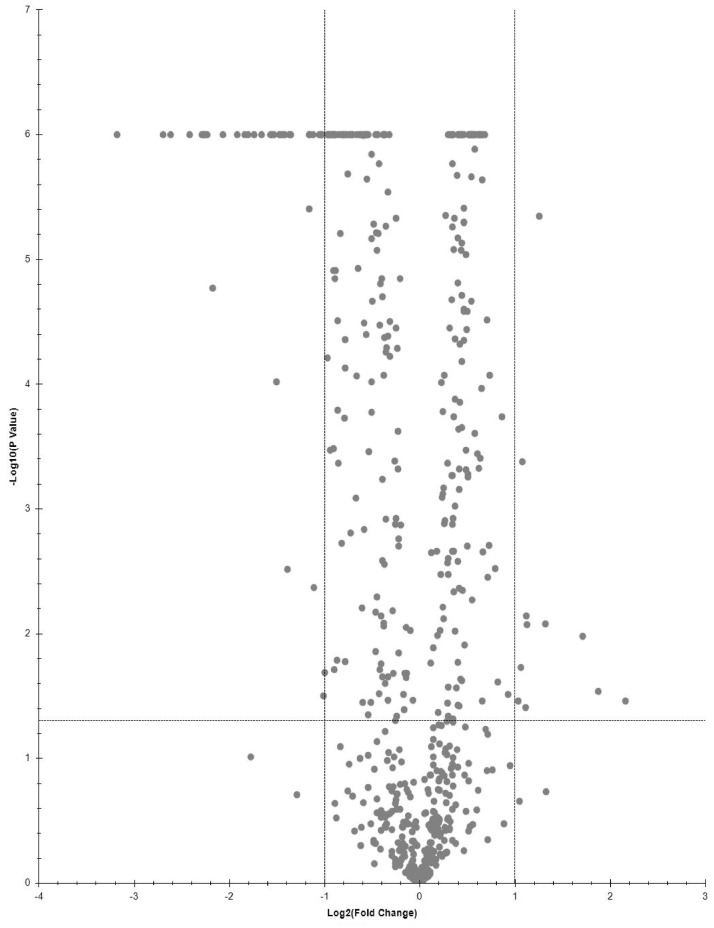
Quantitative proteomics data for cell culture in stationary state: nucleoid to cell lysate.

**Fig. 3:**
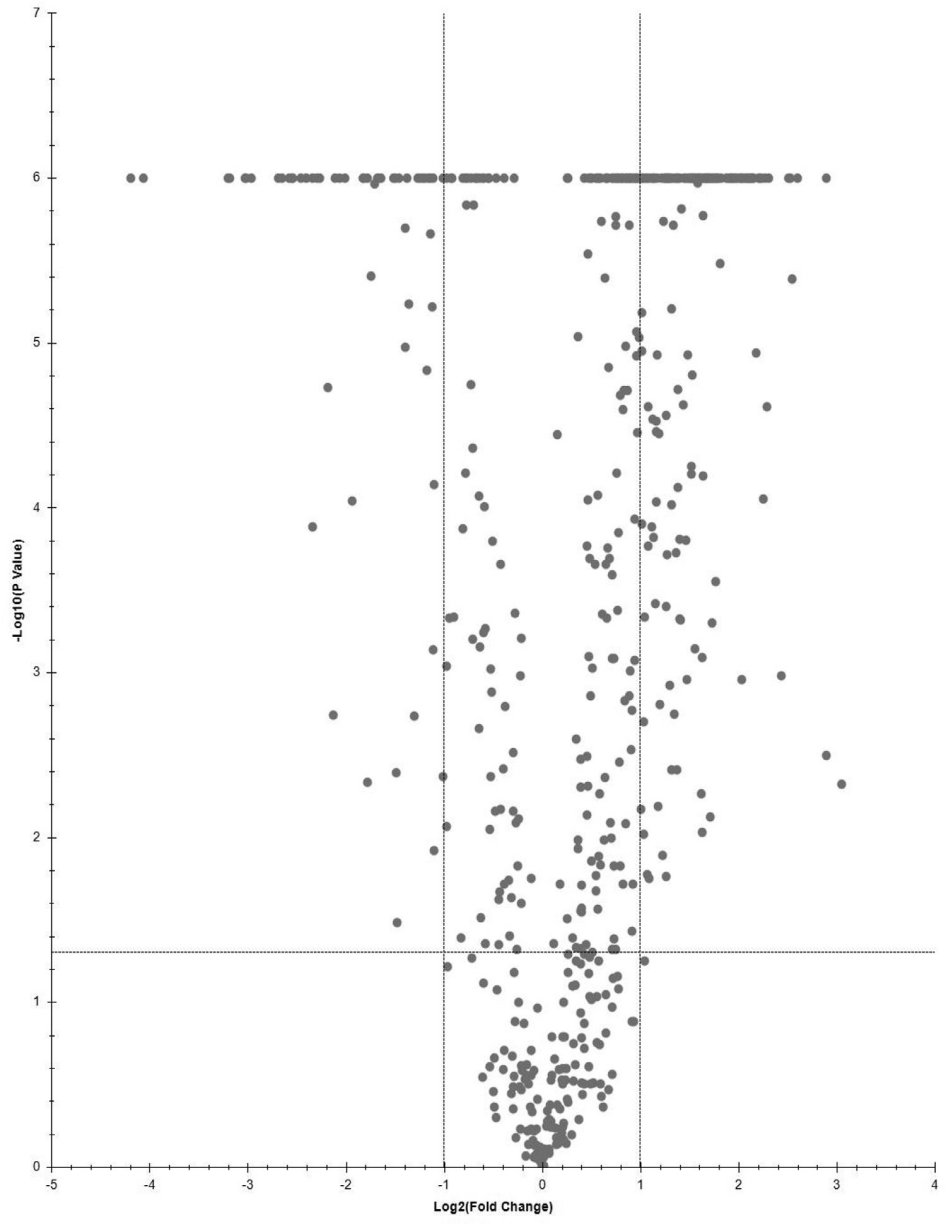
Quantitative proteomics data for nucleoids of cell culture in LPSG to SPSG.

**Fig. 4:**
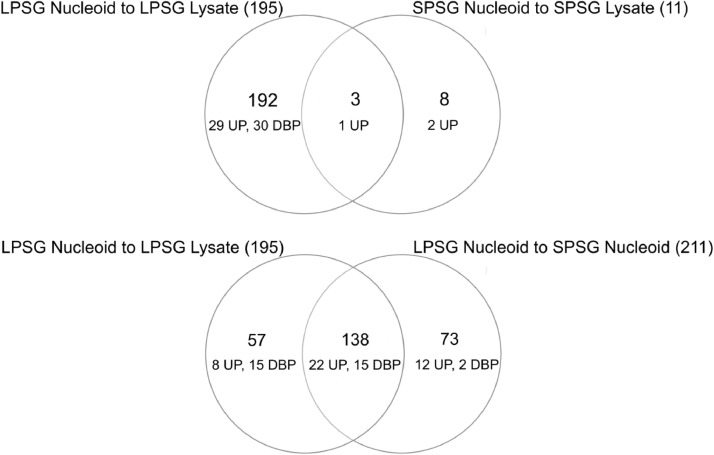
Venn diagrams for quantitative proteomics data, UP - Uncharacterized proteins, DBP - DNA-binding proteins.

Proteins, significantly changing between LPSG and SPSG, are presented on volcano plot (Fig. 1–3). Three sets of abundant (Log2FC > 1, p-value < 0.05) proteins were identified. Two for each LPSG and SPSG nucleoids in comparison to the corresponding cell lysate and one for LPSG nucleoids in comparison to nucleoids isolated from bacteria in SPSG. In total, 195 abundant proteins were identified in LPSG nucleoids and 11 in SPSG. In case of LPSG and SPSG comparison, 211 abundant proteins were identified (Fig. 4).

## 2 Experimental design, materials, and methods

### 2.1 Bacterial strains and cell culture synchronization

*Mycoplasma gallisepticum S6* culture cells were subjected to starvation for 9 h on a liquid medium containing tryptose 20 g/l, Tris 3 g/l, NaCl 5 g/l, KCl 5 g/l in aerobic conditions at pH 7.4 and 37 °C, after that yeast extract (10%, Helicon, Russia), horse serum (20%, Biolot, Russia), glucose 1% (Sigma) and penicillin (Sintez, Russia) with a final concentration 500 units/ml were added. The culture was grown further at 37 °C to logarithmic and stationary growth phases. The LPSG and SPSG culture was taken for nucleoid isolation.

### 2.2 Nucleoid isolation

MG nucleoid fractions were isolated using the method described by Murphy et al. [7] with modification. Cells (30 or 20 ml of culture for logarithmic or stationary phase respectively) were harvested and washed twice by addition of cold washing buffer (50 mM Tris–HCl (Panreac, USA), pH 7.4, 150 mM NaCl and 3 mM MgCl2) and centrifugation at 10,000 g at 4 °C for 10 min. Then the pellets were redispersed in 0.5 ml of Solution A containing 10% sucrose, 10 mM Tris–HCl pH8.2, 100 mM NaCl, 20% sucrose and protease inhibitor cocktail (GE HealthCare, USA). Then, 0.5 ml of solution B (10 mM Tris–HCl, pH8.2, 100 mM NaCl, 10 mM EDTA, 10 mM spermidine (Sigma-Aldrich, USA) and 2% NP-40 (Sigma-Aldrich, USA) was added. After incubation for 10 min, cell lysate was loaded onto a sucrose gradient (10 ml total gradient volume in 15 ml plastic tubes, using gradients with a linear increase from 20 to 60% sucrose in Solution A). The tubes were then centrifuged for 90 min at 10,000 g at 4 °C. A white nucleoid-containing clots were adhered to the tubes walls near the middle of the gradients and were removed from the tubes as the “isolated nucleoids”. Nucleoids were then washed by addition of 1 ml of washing buffer and mixed gently on rotating tube mixer at 4 °C for 20 min. Nucleoids were then centrifuged for 10 min at 15,000 g, 4 °C and the supernatant was discarded.

### 2.3 Tryptic digestion

Sample preparation for proteomic analysis was performed as follows: samples were lysed in a lysis buffer containing 1% sodium deoxycholate (CDNa, Sigma), 100 mM Tris–HCl, pH 8.5 with protease inhibitor cocktail (GE HealthCare) by ultrasonication with a Branson 1510 sonicator at 4 °C within 1 min. Protein concentration was estimated by BCA Assay (Sigma). Aliquots containing 100 mg of protein material were diluted to 1 mg/ml with the lysis buffer and Tris (2-Carboxyethyl) phosphine Hydrochloride (TCEP, Sigma) and chloroacetamide (CAA, Sigma) were added to the final concentrations of 10 and 30 mM respectively. Cys-reduction and alkylation was achieved by 10 min heating of the sample at 80 °C. Proteins were precipitated by addition of 4x volume of acetone and incubation at −20 °C overnight. Protein pellet was washed twice with acetone. Then the pellet was dried and resuspended in 50 ml of 100 mM Tris–HCl, pH 8.5 with 0.1% CDNa in a sonication bath. Trypsin (Promega, USA) was added at a ratio 1:100 w/w to protein amount and incubated at 37 °C overnight. Then the second trypsin portion 1:100 w/w was added, and the sample was incubated for 4 h at 37 °C. Proteolysis was stopped by adding trifluoroacetic acid to 1%. Precipitated CDNa was removed by centrifugation. The samples were analyzed by LC-MS.

### 2.4 DIA lc-ms analysis

LC-MS analysis was carried out on an Ultimate 3000 RSLCnano HPLC system connected to a Fusion Lumos mass spectrometer, controlled by XCalibur software version 4.3.73.11 (Thermo Fisher Scientific). Each sample was injected together with a homemade iRT peptide mixture [8]. Samples were loaded to a 20 × 0.1 mm PepMap C18 5 m trap column (Thermo Fisher Scientific) in the loading buffer (2% ACN, 98% H2O, 0.1% TFA) at 10 µ/min flow and separated at RT in a home-packed 300 × 0.1 mm fused-silica pulled emitter column packed with Reprosil PUR C18AQ 1.9 (Dr. Maisch) [9]. Samples were eluted with a linear gradient of 80% ACN, 19.9% H2O, 0.1% FA (buffer B) in 99.9% H2O, 0.1% FA (solvent A) from 8 to 50% of solvent B in 30 min at 0.5 l/min flow.

In total for each sample type 3 biological replicates with from 2 to 3 technical sample preparation replicates were analyzed. MS data was collected in DDA mode for spectra library generation and in DIA mode for peptide and protein quantitation.

For comprehensive peptide library generation one sample from each biological replicate was run in DDA mode. The samples were run with 3 slightly different DDA methods for better total peptide identification coverage. MS1 parameters were as follows: 120 K resolution, 350–1010 scan range, Standard AGC target and Auto Maximum Injection time. Ions were isolated with 1.6 *m/z* window targeting the highest intensity peaks of +2 to +7 charge, 5 × 104 Intensity Threshold. Dynamic exclusion was set to 20 s. MS2 fragmentation was carried out in HCD mode at 7,5 K resolution with 30% NCE. Mass range was set to Normal, Scan range to Auto, AGC target to Standard. Max injection time was set to 18 ms. Total cycle time was set to 2 s. The second DDA method used 10 ms dynamic exclusion and the third method used 20 s dynamic exclusion but 15 K MS2 resolution and 22 ms max injection time.

All the sample were run in a single LC-MS DIA run. DIA parent ion mass range was from 350 to 1010 *m/z*/ divided into 45 windows 14 Da wide. MS2 resolution was set 7.5 K and Maximum injection time was set to 18 ms. The rest of the parameters were set to default values. (Table 1)

**Table 1:** List of samples.

Num	File	Acquisition	Sample	Case
1	Mg-log-lysate-sucrose-1–(2)_OP-84+iRT_DDA.raw	DDA	OP84	Cell lysate, log phase
2	Mg-log-lysate-sucrose-1–(1)_OP-84+iRT_DIA.raw	DIA	OP84	Cell lysate, log phase
3	Mg-log-lysate-sucrose-1–(2)_OP-85+iRT_DIA.raw	DIA	OP85	Cell lysate, log phase
4	Mg-log-lysate-sucrose-2–(1)_OP-86+iRT_DIA.raw	DIA	OP86	Cell lysate, log phase
5	Mg-log-lysate-sucrose-2–(2)_OP-87+iRT_DIA.raw	DIA	OP87	Cell lysate, log phase
6	Mg-log-lysate-sucrose-3–(1)_OP-88+iRT_DIA.raw	DIA	OP88	Cell lysate, log phase
7	Mg-log-lysate-sucrose-3–(2)_OP-89+iRT_DIA.raw	DIA	OP89	Cell lysate, log phase
8	Mg-log-nucleoid–sucrose-1–(1)_OP-90+iRT_DDA.raw	DDA	OP90	Nucleoid, log phase
9	Mg-log-nucleoid–sucrose-1–(1)_OP-90+iRT_DIA.raw	DIA	OP90	Nucleoid, log phase
10	Mg-log-nucleoid–sucrose-1–(2)_OP-91+iRT_DIA.raw	DIA	OP91	Nucleoid, log phase
11	Mg-log-nucleoid–sucrose-1–(3)_OP-92+iRT_DIA.raw	DIA	OP92	Nucleoid, log phase
12	Mg-log-nucleoid–sucrose-2–(1)_OP-93+iRT_DIA.raw	DIA	OP93	Nucleoid, log phase
13	Mg-log-nucleoid–sucrose-2–(2)_OP-94+iRT_DIA.raw	DIA	OP94	Nucleoid, log phase
14	Mg-log-nucleoid–sucrose-2–(3)_OP-95+iRT_DIA.raw	DIA	OP95	Nucleoid, log phase
15	Mg-log-nucleoid–sucrose-3–(1)_OP-96+iRT_DIA.raw	DIA	OP96	Nucleoid, log phase
16	Mg-log-nucleoid–sucrose-3–(2)_OP-97+iRT_DIA.raw	DIA	OP97	Nucleoid, log phase
17	Mg-log-nucleoid–sucrose-3–(3)_OP-98+iRT_DIA.raw	DIA	OP98	Nucleoid, log phase
18	Mg-stat-lysate-sucrose-1–(2)_OP-108+iRT_2_DDA.raw	DDA	OP108_2	Cell lysate, stationary phase
19	Mg-stat-lysate-sucrose-1–(2)_OP-108+iRT_DDA.raw	DDA	OP108	Cell lysate, stationary phase
20	Mg-stat-lysate-sucrose-1–(1)_OP-108+iRT_DIA.raw	DIA	OP108	Cell lysate, stationary phase
21	Mg-stat-lysate-sucrose-1–(1)_OP-108+iRT_DIA_2.raw	DIA	OP108_2	Cell lysate, stationary phase
22	Mg-stat-lysate-sucrose-1–(2)_OP-109+iRT_DIA.raw	DIA	OP109	Cell lysate, stationary phase
23	Mg-stat-lysate-sucrose-1–(2)_OP-109+iRT_DIA_2.raw	DIA	OP109_2	Cell lysate, stationary phase
24	Mg-stat-lysate-sucrose-2–(1)_OP-110+iRT_DDA.raw	DDA	OP110	Cell lysate, stationary phase
25	Mg-stat-lysate-sucrose-2–(1)_OP-110+iRT_DIA.raw	DIA	OP110	Cell lysate, stationary phase
26	Mg-stat-lysate-sucrose-2–(2)_OP-111+iRT_DIA.raw	DIA	OP111	Cell lysate, stationary phase
27	Mg-stat-lysate-sucrose-3–(1)_OP-112+iRT_DDA.raw	DDA	OP112	Cell lysate, stationary phase
28	Mg-stat-lysate-sucrose-3–(1)_OP-112+iRT_DIA.raw	DIA	OP112	Cell lysate, stationary phase
29	Mg-stat-lysate-sucrose-3–(2)_OP-113+iRT_DIA.raw	DIA	OP113	Cell lysate, stationary phase
30	Mg-stat-nucleoid–sucrose-1–(1)_OP-114+iRT_DDA.raw	DDA	OP114	Nucleoid, stationary phase
31	Mg-stat-nucleoid–sucrose-1–(1)_OP-114+iRT_DIA.raw	DIA	OP114	Nucleoid, stationary phase
32	Mg-stat-nucleoid–sucrose-1–(2)_OP-115+iRT_DIA.raw	DIA	OP115	Nucleoid, stationary phase
33	Mg-stat-nucleoid–sucrose-1–(3)_OP-116+iRT_DIA.raw	DIA	OP116	Nucleoid, stationary phase
34	Mg-stat-nucleoid–sucrose-2–(1)_OP-117+iRT_DDA.raw	DDA	OP117	Nucleoid, stationary phase
35	Mg-stat-nucleoid–sucrose-2–(1)_OP-117+iRT_DIA.raw	DIA	OP117	Nucleoid, stationary phase
36	Mg-stat-nucleoid–sucrose-2–(2)_OP-118+iRT_DIA.raw	DIA	OP118	Nucleoid, stationary phase
37	Mg-stat-nucleoid–sucrose-2–(3)_OP-119+iRT_DIA.raw	DIA	OP119	Nucleoid, stationary phase
38	Mg-stat-nucleoid–sucrose-3–(1)_OP-120+iRT_DDA.raw	DDA	OP120	Nucleoid, stationary phase
39	Mg-stat-nucleoid–sucrose-3–(1)_OP-120+iRT_DIA.raw	DIA	OP120	Nucleoid, stationary phase
40	Mg-stat-nucleoid–sucrose-3–(2)_OP-121+iRT_DIA.raw	DIA	OP121	Nucleoid, stationary phase
41	Mg-stat-nucleoid–sucrose-3–(3)_OP-122+iRT_DIA.raw	DIA	OP122	Nucleoid, stationary phase

### 2.5 Data processing protocol

Identification of DDA files was performed with MaxQuant 1.6.6.0 Software with default settings against the *M. gallisepticum S6* Uniprot reference database. The resulting list of peptides was used to create a spectral library in Skyline Software.

Further analysis of DIA files was performed in Skyline software using default DIA protocol. Retention times were aligned using built-in iRT calculator and DDA files. The same *M. gallisepticum S6* Uniprot database was used to create a transition list. Quantitative analysis was also performed using the default quantification protocol. The resulting quantification data was normalized by equalizing the run medians.

## Declaration of Competing Interest

The authors declare that they have no known competing financial interests or personal relationships which have, or could be perceived to have, influenced the work reported in this article.

## Appendix Supplementary materials

Image, application 1

Image, application 2
